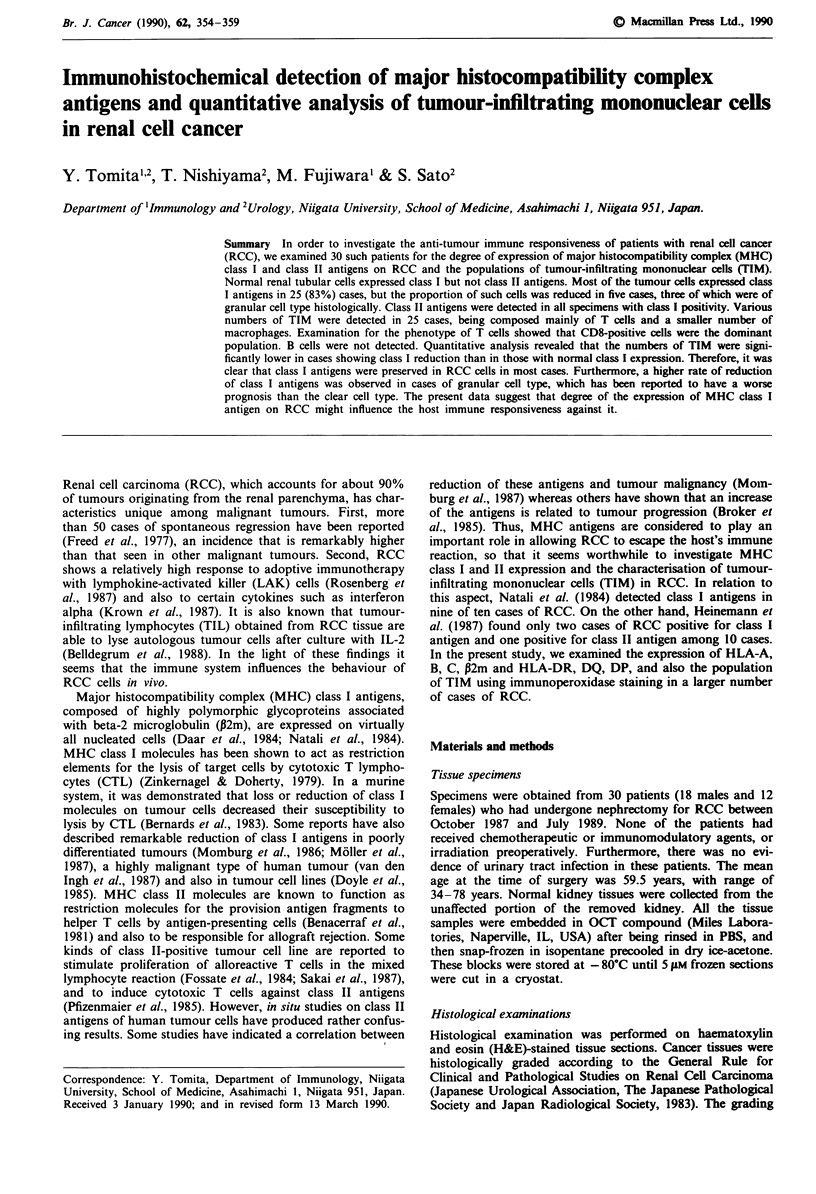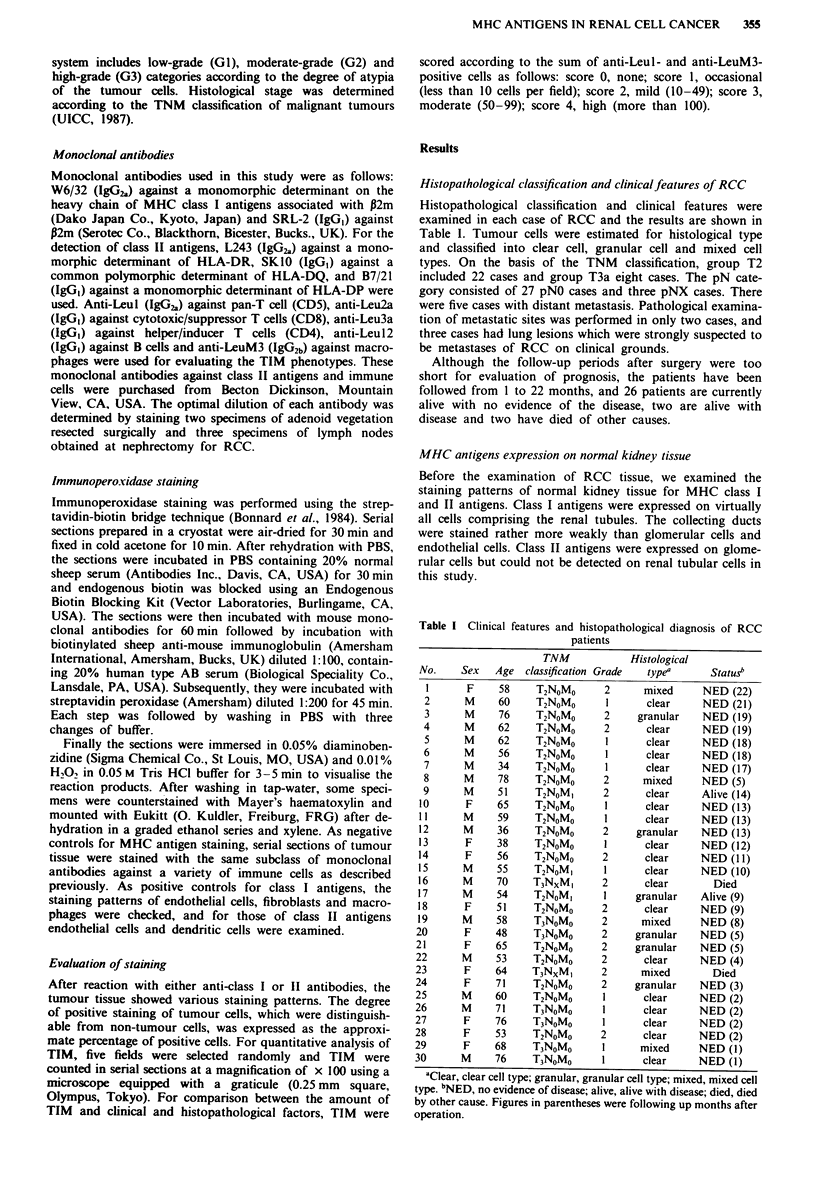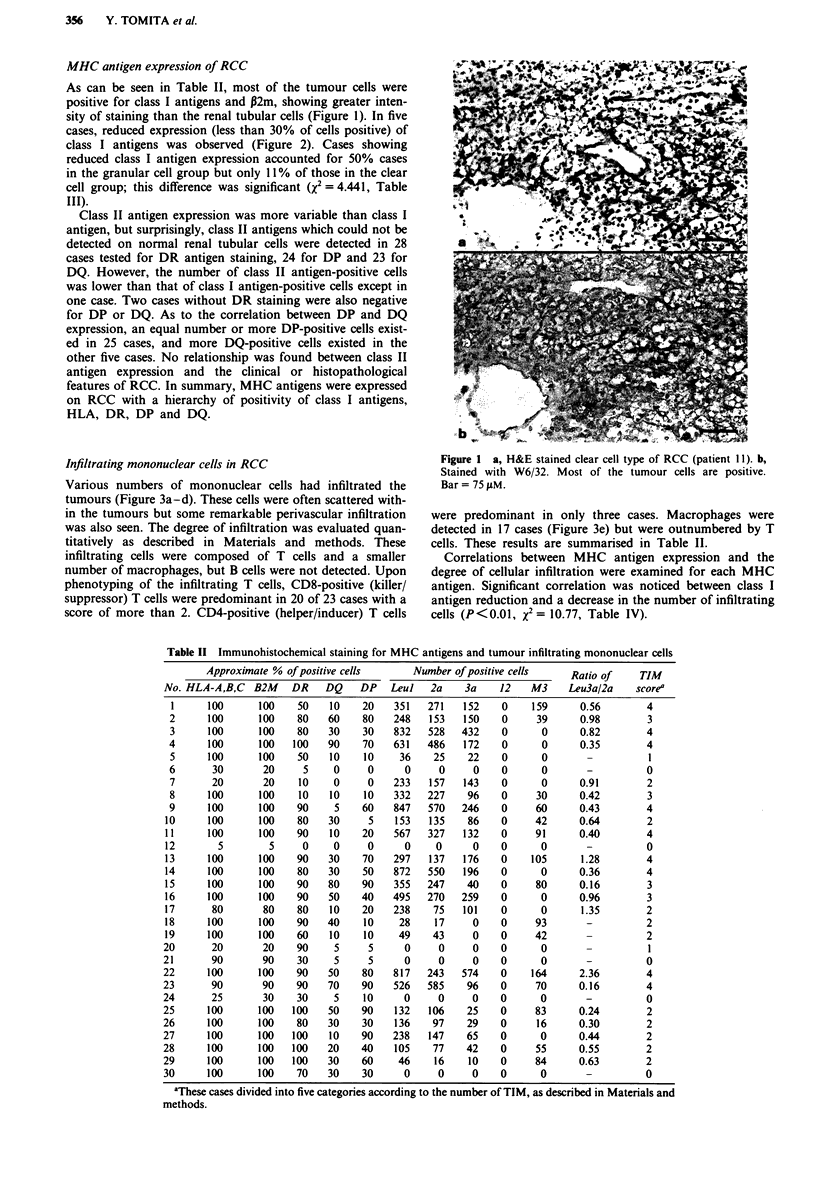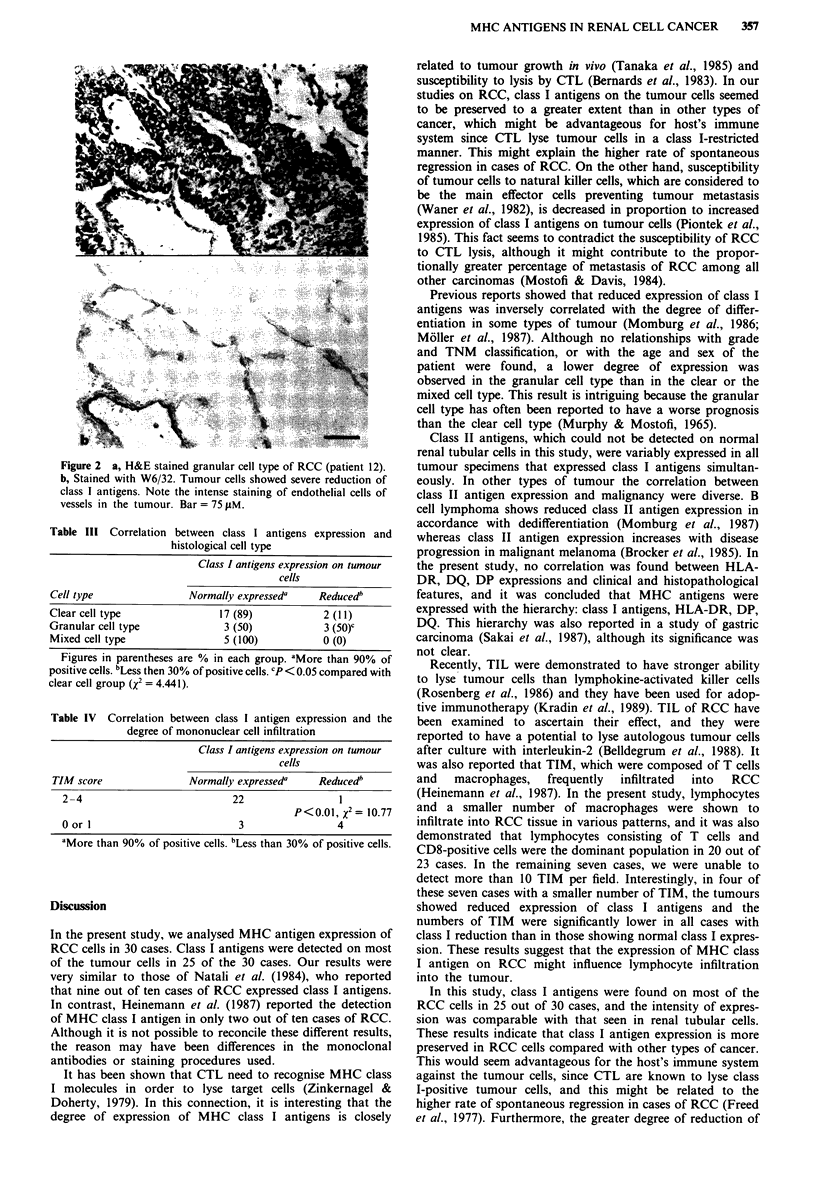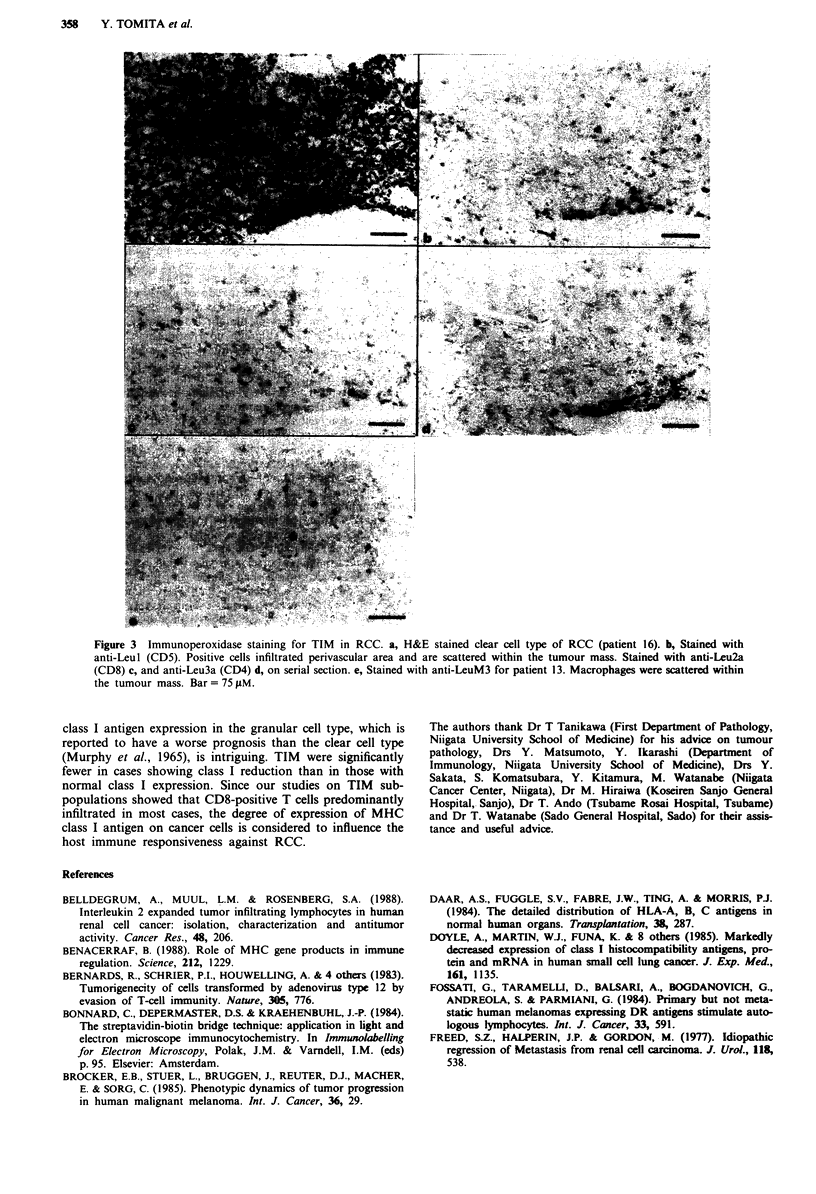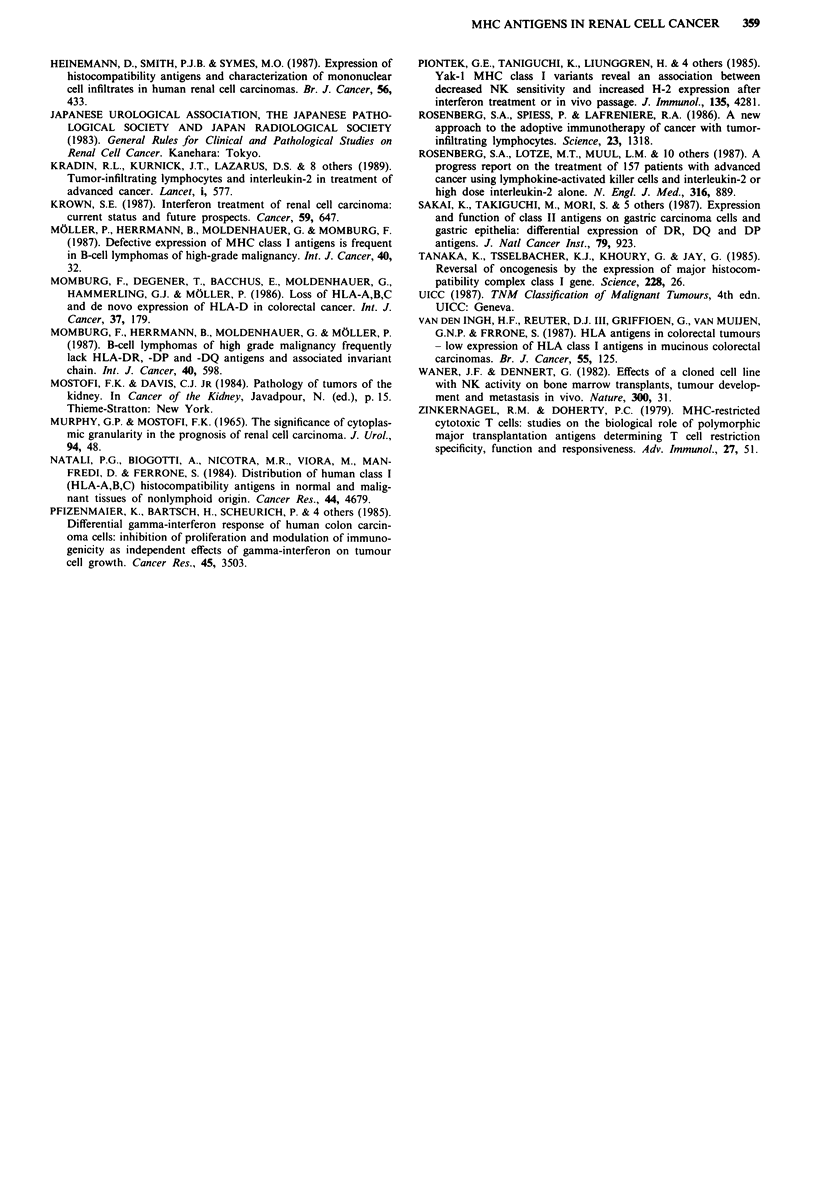# Immunohistochemical detection of major histocompatibility complex antigens and quantitative analysis of tumour-infiltrating mononuclear cells in renal cell cancer.

**DOI:** 10.1038/bjc.1990.296

**Published:** 1990-09

**Authors:** Y. Tomita, T. Nishiyama, M. Fujiwara, S. Sato

**Affiliations:** Department of Immunology, Niigata University, School of Medicine, Japan.

## Abstract

**Images:**


*Br. J. Cancer* (1990), **62**, 354–359 © Macmillan Press Ltd., 1990

### Immunohistochemical detection of major histocompatibility complex antigens and quantitative analysis of tumour-infiltrating mononuclear cells in renal cell cancer

Y. Tomita[1,2], T. Nishiyama[2], M. Fujiwara[1] & S. Sato[2]

*Department of [1]Immunology and [2]Urology, Niigata University, School of Medicine, Asahimachi 1, Niigata 951, Japan.*

**Summary** In order to investigate the anti-tumour immune responsiveness of patients with renal cell cancer (RCC), we examined 30 such patients for the degree of expression of major histocompatibility complex (MHC) class I and class II antigens on RCC and the populations of tumour-infiltrating mononuclear cells (TIM). Normal renal tubular cells expressed class I but not class II antigens. Most of the tumour cells expressed class I antigens in 25 (83%) cases, but the proportion of such cells was reduced in five cases, three of which were of granular cell type histologically. Class II antigens were detected in all specimens with class I positivity. Various numbers of TIM were detected in 25 cases, being composed mainly of T cells and a smaller number of macrophages. Examination for the phenotype of T cells showed that CD8-positive cells were the dominant population. B cells were not detected. Quantitative analysis revealed that the numbers of TIM were significantly lower in cases showing class I reduction than in those with normal class I expression. Therefore, it was clear that class I antigens were preserved in RCC cells in most cases. Furthermore, a higher rate of reduction of class I antigens was observed in cases of granular cell type, which has been reported to have a worse prognosis than the clear cell type. The present data suggest that degree of the expression of MHC class I antigen on RCC might influence the host immune responsiveness against it.

Renal cell carcinoma (RCC), which accounts for about 90% of tumours originating from the renal parenchyma, has characteristics unique among malignant tumours. First, more than 50 cases of spontaneous regression have been reported (Freed *et al.*, 1977), an incidence that is remarkably higher than that seen in other malignant tumours. Second, RCC shows a relatively high response to adoptive immunotherapy with lymphokine-activated killer (LAK) cells (Rosenberg *et al.*, 1987) and also to certain cytokines such as interferon alpha (Krown *et al.*, 1987). It is also known that tumour-infiltrating lymphocytes (TIL) obtained from RCC tissue are able to lyse autologous tumour cells after culture with IL-2 (Belldegrum *et al.*, 1988). In the light of these findings it seems that the immune system influences the behaviour of RCC cells *in vivo*.

Major histocompatibility complex (MHC) class I antigens, composed of highly polymorphic glycoproteins associated with beta-2 microglobulin (β2m), are expressed on virtually all nucleated cells (Daar *et al.*, 1984; Natali *et al.*, 1984). MHC class I molecules has been shown to act as restriction elements for the lysis of target cells by cytotoxic T lymphocytes (CTL) (Zinkernagel & Doherty, 1979). In a murine system, it was demonstrated that loss or reduction of class I molecules on tumour cells decreased their susceptibility to lysis by CTL (Bernards *et al.*, 1983). Some reports have also described remarkable reduction of class I antigens in poorly differentiated tumours (Momburg *et al.*, 1986; Möller *et al.*, 1987), a highly malignant type of human tumour (van den Ingh *et al.*, 1987) and also in tumour cell lines (Doyle *et al.*, 1985). MHC class II molecules are known to function as restriction molecules for the provision antigen fragments to helper T cells by antigen-presenting cells (Benacerraf *et al.*, 1981) and also to be responsible for allograft rejection. Some kinds of class II-positive tumour cell line are reported to stimulate proliferation of alloreactive T cells in the mixed lymphocyte reaction (Fossate *et al.*, 1984; Sakai *et al.*, 1987), and to induce cytotoxic T cells against class II antigens (Pfizenmaier *et al.*, 1985). However, *in situ* studies on class II antigens of human tumour cells have produced rather confusing results. Some studies have indicated a correlation between reduction of these antigens and tumour malignancy (Momburg *et al.*, 1987) whereas others have shown that an increase of the antigens is related to tumour progression (Broker *et al.*, 1985). Thus, MHC antigens are considered to play an important role in allowing RCC to escape the host's immune reaction, so that it seems worthwhile to investigate MHC class I and II expression and the characterisation of tumour-infiltrating mononuclear cells (TIM) in RCC. In relation to this aspect, Natali *et al.* (1984) detected class I antigens in nine of ten cases of RCC. On the other hand, Heinemann *et al.* (1987) found only two cases of RCC positive for class I antigen and one positive for class II antigen among 10 cases. In the present study, we examined the expression of HLA-A, B, C, β2m and HLA-DR, DQ, DP, and also the population of TIM using immunoperoxidase staining in a larger number of cases of RCC.

### Materials and methods

### *Tissue specimens*

Specimens were obtained from 30 patients (18 males and 12 females) who had undergone nephrectomy for RCC between October 1987 and July 1989. None of the patients had received chemotherapeutic or immunomodulatory agents, or irradiation preoperatively. Furthermore, there was no evidence of urinary tract infection in these patients. The mean age at the time of surgery was 59.5 years, with range of 34–78 years. Normal kidney tissues were collected from the unaffected portion of the removed kidney. All the tissue samples were embedded in OCT compound (Miles Laboratories, Naperville, IL, USA) after being rinsed in PBS, and then snap-frozen in isopentane precooled in dry ice-acetone. These blocks were stored at −80°C until 5 μM frozen sections were cut in a cryostat.

### *Histological examinations*

Histological examination was performed on haematoxylin and eosin (H&E)-stained tissue sections. Cancer tissues were histologically graded according to the General Rule for Clinical and Pathological Studies on Renal Cell Carcinoma (Japanese Urological Association, The Japanese Pathological Society and Japan Radiological Society, 1983). The grading

Correspondence: Y. Tomita, Department of Immunology, Niigata University, School of Medicine, Asahimachi 1, Niigata 951, Japan.
Received 3 January 1990; and in revised form 13 March 1990.

system includes low-grade (G1), moderate-grade (G2) and high-grade (G3) categories according to the degree of atypia of the tumour cells. Histological stage was determined according to the TNM classification of malignant tumours (UICC, 1987).

### *Monoclonal antibodies*

Monoclonal antibodies used in this study were as follows: W6/32 ($IgG_{2a}$) against a monomorphic determinant on the heavy chain of MHC class I antigens associated with β2m (Dako Japan Co., Kyoto, Japan) and SRL-2 ($IgG_1$) against β2m (Serotec Co., Blackthorn, Bicester, Bucks., UK). For the detection of class II antigens, L243 ($IgG_{2a}$) against a monomorphic determinant of HLA-DR, SK10 ($IgG_1$) against a common polymorphic determinant of HLA-DQ, and B7/21 ($IgG_1$) against a monomorphic determinant of HLA-DP were used. Anti-Leu1 ($IgG_{2a}$) against pan-T cell (CD5), anti-Leu2a ($IgG_1$) against cytotoxic/suppressor T cells (CD8), anti-Leu3a ($IgG_1$) against helper/inducer T cells (CD4), anti-Leu12 ($IgG_1$) against B cells and anti-LeuM3 ($IgG_{2b}$) against macrophages were used for evaluating the TIM phenotypes. These monoclonal antibodies against class II antigens and immune cells were purchased from Becton Dickinson, Mountain View, CA, USA. The optimal dilution of each antibody was determined by staining two specimens of adenoid vegetation resected surgically and three specimens of lymph nodes obtained at nephrectomy for RCC.

### *Immunoperoxidase staining*

Immunoperoxidase staining was performed using the streptavidin-biotin bridge technique (Bonnard *et al.*, 1984). Serial sections prepared in a cryostat were air-dried for 30 min and fixed in cold acetone for 10 min. After rehydration with PBS, the sections were incubated in PBS containing 20% normal sheep serum (Antibodies Inc., Davis, CA, USA) for 30 min and endogenous biotin was blocked using an Endogenous Biotin Blocking Kit (Vector Laboratories, Burlingame, CA, USA). The sections were then incubated with mouse monoclonal antibodies for 60 min followed by incubation with biotinylated sheep anti-mouse immunoglobulin (Amersham International, Amersham, Bucks, UK) diluted 1:100, containing 20% human type AB serum (Biological Speciality Co., Lansdale, PA, USA). Subsequently, they were incubated with streptavidin peroxidase (Amersham) diluted 1:200 for 45 min. Each step was followed by washing in PBS with three changes of buffer.

Finally the sections were immersed in 0.05% diaminobenzidine (Sigma Chemical Co., St Louis, MO, USA) and 0.01% $H_2O_2$ in 0.05 M Tris HCl buffer for 3–5 min to visualise the reaction products. After washing in tap-water, some specimens were counterstained with Mayer's haematoxylin and mounted with Eukitt (O. Kuldler, Freiburg, FRG) after dehydration in a graded ethanol series and xylene. As negative controls for MHC antigen staining, serial sections of tumour tissue were stained with the same subclass of monoclonal antibodies against a variety of immune cells as described previously. As positive controls for class I antigens, the staining patterns of endothelial cells, fibroblasts and macrophages were checked, and for those of class II antigens endothelial cells and dendritic cells were examined.

### *Evaluation of staining*

After reaction with either anti-class I or II antibodies, the tumour tissue showed various staining patterns. The degree of positive staining of tumour cells, which were distinguishable from non-tumour cells, was expressed as the approximate percentage of positive cells. For quantitative analysis of TIM, five fields were selected randomly and TIM were counted in serial sections at a magnification of × 100 using a microscope equipped with a graticule (0.25 mm square, Olympus, Tokyo). For comparison between the amount of TIM and clinical and histopathological factors, TIM were scored according to the sum of anti-Leu1- and anti-LeuM3-positive cells as follows: score 0, none; score 1, occasional (less than 10 cells per field); score 2, mild (10–49); score 3, moderate (50–99); score 4, high (more than 100).

### Results

### *Histopathological classification and clinical features of RCC*

Histopathological classification and clinical features were examined in each case of RCC and the results are shown in Table I. Tumour cells were estimated for histological type and classified into clear cell, granular cell and mixed cell types. On the basis of the TNM classification, group T2 included 22 cases and group T3a eight cases. The pN category consisted of 27 pN0 cases and three pNX cases. There were five cases with distant metastasis. Pathological examination of metastatic sites was performed in only two cases, and three cases had lung lesions which were strongly suspected to be metastases of RCC on clinical grounds.

Although the follow-up periods after surgery were too short for evaluation of prognosis, the patients have been followed from 1 to 22 months, and 26 patients are currently alive with no evidence of the disease, two are alive with disease and two have died of other causes.

### *MHC antigens expression on normal kidney tissue*

Before the examination of RCC tissue, we examined the staining patterns of normal kidney tissue for MHC class I and II antigens. Class I antigens were expressed on virtually all cells comprising the renal tubules. The collecting ducts were stained rather more weakly than glomerular cells and endothelial cells. Class II antigens were expressed on glomerular cells but could not be detected on renal tubular cells in this study.

**Table I** Clinical features and histopathological diagnosis of RCC patients

| *No.* | *Sex* | *Age* | *TNM classification* | *Grade* | *Histological type*[a] | *Status*[b] |
|---|---|---|---|---|---|---|
| 1 | F | 58 | $T_2N_0M_0$ | 2 | mixed | NED (22) |
| 2 | M | 60 | $T_2N_0M_0$ | 1 | clear | NED (21) |
| 3 | M | 76 | $T_2N_0M_0$ | 2 | granular | NED (19) |
| 4 | M | 62 | $T_2N_0M_0$ | 2 | clear | NED (19) |
| 5 | M | 62 | $T_2N_0M_0$ | 1 | clear | NED (18) |
| 6 | M | 56 | $T_2N_0M_0$ | 1 | clear | NED (18) |
| 7 | M | 34 | $T_2N_0M_0$ | 1 | clear | NED (17) |
| 8 | M | 78 | $T_2N_0M_0$ | 2 | mixed | NED (5) |
| 9 | M | 51 | $T_2N_0M_1$ | 2 | clear | Alive (14) |
| 10 | F | 65 | $T_2N_0M_0$ | 1 | clear | NED (13) |
| 11 | M | 59 | $T_2N_0M_0$ | 1 | clear | NED (13) |
| 12 | M | 36 | $T_2N_0M_0$ | 2 | granular | NED (13) |
| 13 | F | 38 | $T_2N_0M_0$ | 1 | clear | NED (12) |
| 14 | F | 56 | $T_2N_0M_0$ | 2 | clear | NED (11) |
| 15 | M | 55 | $T_2N_0M_1$ | 1 | clear | NED (10) |
| 16 | M | 70 | $T_3N_XM_1$ | 2 | clear | Died |
| 17 | M | 54 | $T_2N_0M_1$ | 1 | granular | Alive (9) |
| 18 | F | 51 | $T_2N_0M_0$ | 2 | clear | NED (9) |
| 19 | M | 58 | $T_3N_0M_0$ | 2 | mixed | NED (8) |
| 20 | F | 48 | $T_3N_0M_0$ | 2 | granular | NED (5) |
| 21 | F | 65 | $T_2N_0M_0$ | 2 | granular | NED (5) |
| 22 | M | 53 | $T_2N_0M_0$ | 2 | clear | NED (4) |
| 23 | F | 64 | $T_3N_XM_1$ | 2 | mixed | Died |
| 24 | F | 71 | $T_2N_0M_0$ | 2 | granular | NED (3) |
| 25 | M | 60 | $T_2N_0M_0$ | 1 | clear | NED (2) |
| 26 | M | 71 | $T_3N_0M_0$ | 1 | clear | NED (2) |
| 27 | F | 76 | $T_3N_0M_0$ | 1 | clear | NED (2) |
| 28 | F | 53 | $T_2N_0M_0$ | 2 | clear | NED (2) |
| 29 | F | 68 | $T_3N_0M_0$ | 1 | mixed | NED (1) |
| 30 | M | 76 | $T_3N_0M_0$ | 1 | clear | NED (1) |

[a]Clear, clear cell type; granular, granular cell type; mixed, mixed cell type. [b]NED, no evidence of disease; alive, alive with disease; died, died by other cause. Figures in parentheses were following up months after operation.

*MHC antigen expression of RCC*

As can be seen in Table II, most of the tumour cells were positive for class I antigens and β2m, showing greater intensity of staining than the renal tubular cells (Figure 1). In five cases, reduced expression (less than 30% of cells positive) of class I antigens was observed (Figure 2). Cases showing reduced class I antigen expression accounted for 50% cases in the granular cell group but only 11% of those in the clear cell group; this difference was significant ($\chi^2 = 4.441$, Table III).

Class II antigen expression was more variable than class I antigen, but surprisingly, class II antigens which could not be detected on normal renal tubular cells were detected in 28 cases tested for DR antigen staining, 24 for DP and 23 for DQ. However, the number of class II antigen-positive cells was lower than that of class I antigen-positive cells except in one case. Two cases without DR staining were also negative for DP or DQ. As to the correlation between DP and DQ expression, an equal number or more DP-positive cells existed in 25 cases, and more DQ-positive cells existed in the other five cases. No relationship was found between class II antigen expression and the clinical or histopathological features of RCC. In summary, MHC antigens were expressed on RCC with a hierarchy of positivity of class I antigens, HLA, DR, DP and DQ.

**Figure 1** **a**, H&E stained clear cell type of RCC (patient 11). **b**, Stained with W6/32. Most of the tumour cells are positive. Bar = 75 μM.

*Infiltrating mononuclear cells in RCC*

Various numbers of mononuclear cells had infiltrated the tumours (Figure 3a–d). These cells were often scattered within the tumours but some remarkable perivascular infiltration was also seen. The degree of infiltration was evaluated quantitatively as described in Materials and methods. These infiltrating cells were composed of T cells and a smaller number of macrophages, but B cells were not detected. Upon phenotyping of the infiltrating T cells, CD8-positive (killer/suppressor) T cells were predominant in 20 of 23 cases with a score of more than 2. CD4-positive (helper/inducer) T cells were predominant in only three cases. Macrophages were detected in 17 cases (Figure 3e) but were outnumbered by T cells. These results are summarised in Table II.

Correlations between MHC antigen expression and the degree of cellular infiltration were examined for each MHC antigen. Significant correlation was noticed between class I antigen reduction and a decrease in the number of infiltrating cells ($P<0.01$, $\chi^2 = 10.77$, Table IV).

**Table II** Immunohistochemical staining for MHC antigens and tumour infiltrating mononuclear cells

| | *Approximate % of positive cells* | | | | | *Number of positive cells* | | | | | | |
|---|---|---|---|---|---|---|---|---|---|---|---|---|
| *No.* | *HLA-A,B,C* | *B2M* | *DR* | *DQ* | *DP* | *Leu1* | *2a* | *3a* | *12* | *M3* | *Ratio of Leu3a/2a* | *TIM score*[a] |
| 1 | 100 | 100 | 50 | 10 | 20 | 351 | 271 | 152 | 0 | 159 | 0.56 | 4 |
| 2 | 100 | 100 | 80 | 60 | 80 | 248 | 153 | 150 | 0 | 39 | 0.98 | 3 |
| 3 | 100 | 100 | 80 | 30 | 30 | 832 | 528 | 432 | 0 | 0 | 0.82 | 4 |
| 4 | 100 | 100 | 100 | 90 | 70 | 631 | 486 | 172 | 0 | 0 | 0.35 | 4 |
| 5 | 100 | 100 | 50 | 10 | 10 | 36 | 25 | 22 | 0 | 0 | – | 1 |
| 6 | 30 | 20 | 5 | 0 | 0 | 0 | 0 | 0 | 0 | 0 | – | 0 |
| 7 | 20 | 20 | 10 | 0 | 0 | 233 | 157 | 143 | 0 | 0 | 0.91 | 2 |
| 8 | 100 | 100 | 10 | 10 | 10 | 332 | 227 | 96 | 0 | 30 | 0.42 | 3 |
| 9 | 100 | 100 | 90 | 5 | 60 | 847 | 570 | 246 | 0 | 60 | 0.43 | 4 |
| 10 | 100 | 100 | 80 | 30 | 5 | 153 | 135 | 86 | 0 | 42 | 0.64 | 2 |
| 11 | 100 | 100 | 90 | 10 | 20 | 567 | 327 | 132 | 0 | 91 | 0.40 | 4 |
| 12 | 5 | 5 | 0 | 0 | 0 | 0 | 0 | 0 | 0 | 0 | – | 0 |
| 13 | 100 | 100 | 90 | 30 | 70 | 297 | 137 | 176 | 0 | 105 | 1.28 | 4 |
| 14 | 100 | 100 | 80 | 30 | 50 | 872 | 550 | 196 | 0 | 0 | 0.36 | 4 |
| 15 | 100 | 100 | 90 | 80 | 90 | 355 | 247 | 40 | 0 | 80 | 0.16 | 3 |
| 16 | 100 | 100 | 90 | 50 | 40 | 495 | 270 | 259 | 0 | 0 | 0.96 | 3 |
| 17 | 80 | 80 | 80 | 10 | 20 | 238 | 75 | 101 | 0 | 0 | 1.35 | 2 |
| 18 | 100 | 100 | 90 | 40 | 10 | 28 | 17 | 0 | 0 | 93 | – | 2 |
| 19 | 100 | 100 | 60 | 10 | 10 | 49 | 43 | 0 | 0 | 42 | – | 2 |
| 20 | 20 | 20 | 90 | 5 | 5 | 0 | 0 | 0 | 0 | 0 | – | 1 |
| 21 | 90 | 90 | 30 | 5 | 5 | 0 | 0 | 0 | 0 | 0 | – | 0 |
| 22 | 100 | 100 | 90 | 50 | 80 | 817 | 243 | 574 | 0 | 164 | 2.36 | 4 |
| 23 | 90 | 90 | 90 | 70 | 90 | 526 | 585 | 96 | 0 | 70 | 0.16 | 4 |
| 24 | 25 | 30 | 30 | 5 | 10 | 0 | 0 | 0 | 0 | 0 | – | 0 |
| 25 | 100 | 100 | 100 | 50 | 90 | 132 | 106 | 25 | 0 | 83 | 0.24 | 2 |
| 26 | 100 | 100 | 80 | 30 | 30 | 136 | 97 | 29 | 0 | 16 | 0.30 | 2 |
| 27 | 100 | 100 | 100 | 10 | 90 | 238 | 147 | 65 | 0 | 0 | 0.44 | 2 |
| 28 | 100 | 100 | 100 | 20 | 40 | 105 | 77 | 42 | 0 | 55 | 0.55 | 2 |
| 29 | 100 | 100 | 100 | 30 | 60 | 46 | 16 | 10 | 0 | 84 | 0.63 | 2 |
| 30 | 100 | 100 | 70 | 30 | 30 | 0 | 0 | 0 | 0 | 0 | – | 0 |

[a]These cases divided into five categories according to the number of TIM, as described in Materials and methods.

**Figure 2** **a**, H&E stained granular cell type of RCC (patient 12). **b**, Stained with W6/32. Tumour cells showed severe reduction of class I antigens. Note the intense staining of endothelial cells of vessels in the tumour. Bar = 75 μM.

**Table III** Correlation between class I antigens expression and histological cell type

| | *Class I antigens expression on tumour cells* | |
|---|---|---|
| *Cell type* | *Normally expressed*[a] | *Reduced*[b] |
| Clear cell type | 17 (89) | 2 (11) |
| Granular cell type | 3 (50) | 3 (50)[c] |
| Mixed cell type | 5 (100) | 0 (0) |

Figures in parentheses are % in each group. [a]More than 90% of positive cells. [b]Less then 30% of positive cells. [c]$P < 0.05$ compared with clear cell group ($\chi^2 = 4.441$).

**Table IV** Correlation between class I antigen expression and the degree of mononuclear cell infiltration

| | *Class I antigens expression on tumour cells* | |
|---|---|---|
| *TIM score* | *Normally expressed*[a] | *Reduced*[b] |
| 2–4 | 22 | 1 |
| | | $P < 0.01$, $\chi^2 = 10.77$ |
| 0 or 1 | 3 | 4 |

[a]More than 90% of positive cells. [b]Less than 30% of positive cells.

### Discussion

In the present study, we analysed MHC antigen expression of RCC cells in 30 cases. Class I antigens were detected on most of the tumour cells in 25 of the 30 cases. Our results were very similar to those of Natali *et al.* (1984), who reported that nine out of ten cases of RCC expressed class I antigens. In contrast, Heinemann *et al.* (1987) reported the detection of MHC class I antigen in only two out of ten cases of RCC. Although it is not possible to reconcile these different results, the reason may have been differences in the monoclonal antibodies or staining procedures used.

It has been shown that CTL need to recognise MHC class I molecules in order to lyse target cells (Zinkernagel & Doherty, 1979). In this connection, it is interesting that the degree of expression of MHC class I antigens is closely related to tumour growth *in vivo* (Tanaka *et al.*, 1985) and susceptibility to lysis by CTL (Bernards *et al.*, 1983). In our studies on RCC, class I antigens on the tumour cells seemed to be preserved to a greater extent than in other types of cancer, which might be advantageous for host's immune system since CTL lyse tumour cells in a class I-restricted manner. This might explain the higher rate of spontaneous regression in cases of RCC. On the other hand, susceptibility of tumour cells to natural killer cells, which are considered to be the main effector cells preventing tumour metastasis (Waner *et al.*, 1982), is decreased in proportion to increased expression of class I antigens on tumour cells (Piontek *et al.*, 1985). This fact seems to contradict the susceptibility of RCC to CTL lysis, although it might contribute to the proportionally greater percentage of metastasis of RCC among all other carcinomas (Mostofi & Davis, 1984).

Previous reports showed that reduced expression of class I antigens was inversely correlated with the degree of differentiation in some types of tumour (Momburg *et al.*, 1986; Möller *et al.*, 1987). Although no relationships with grade and TNM classification, or with the age and sex of the patient were found, a lower degree of expression was observed in the granular cell type than in the clear or the mixed cell type. This result is intriguing because the granular cell type has often been reported to have a worse prognosis than the clear cell type (Murphy & Mostofi, 1965).

Class II antigens, which could not be detected on normal renal tubular cells in this study, were variably expressed in all tumour specimens that expressed class I antigens simultaneously. In other types of tumour the correlation between class II antigen expression and malignancy were diverse. B cell lymphoma shows reduced class II antigen expression in accordance with dedifferentiation (Momburg *et al.*, 1987) whereas class II antigen expression increases with disease progression in malignant melanoma (Brocker *et al.*, 1985). In the present study, no correlation was found between HLA-DR, DQ, DP expressions and clinical and histopathological features, and it was concluded that MHC antigens were expressed with the hierarchy: class I antigens, HLA-DR, DP, DQ. This hierarchy was also reported in a study of gastric carcinoma (Sakai *et al.*, 1987), although its significance was not clear.

Recently, TIL were demonstrated to have stronger ability to lyse tumour cells than lymphokine-activated killer cells (Rosenberg *et al.*, 1986) and they have been used for adoptive immunotherapy (Kradin *et al.*, 1989). TIL of RCC have been examined to ascertain their effect, and they were reported to have a potential to lyse autologous tumour cells after culture with interleukin-2 (Belldegrum *et al.*, 1988). It was also reported that TIM, which were composed of T cells and macrophages, frequently infiltrated into RCC (Heinemann *et al.*, 1987). In the present study, lymphocytes and a smaller number of macrophages were shown to infiltrate into RCC tissue in various patterns, and it was also demonstrated that lymphocytes consisting of T cells and CD8-positive cells were the dominant population in 20 out of 23 cases. In the remaining seven cases, we were unable to detect more than 10 TIM per field. Interestingly, in four of these seven cases with a smaller number of TIM, the tumours showed reduced expression of class I antigens and the numbers of TIM were significantly lower in all cases with class I reduction than in those showing normal class I expression. These results suggest that the expression of MHC class I antigen on RCC might influence lymphocyte infiltration into the tumour.

In this study, class I antigens were found on most of the RCC cells in 25 out of 30 cases, and the intensity of expression was comparable with that seen in renal tubular cells. These results indicate that class I antigen expression is more preserved in RCC cells compared with other types of cancer. This would seem advantageous for the host's immune system against the tumour cells, since CTL are known to lyse class I-positive tumour cells, and this might be related to the higher rate of spontaneous regression in cases of RCC (Freed *et al.*, 1977). Furthermore, the greater degree of reduction of

**Figure 3** Immunoperoxidase staining for TIM in RCC. **a**, H&E stained clear cell type of RCC (patient 16). **b**, Stained with anti-Leu1 (CD5). Positive cells infiltrated perivascular area and are scattered within the tumour mass. Stained with anti-Leu2a (CD8) **c**, and anti-Leu3a (CD4) **d**, on serial section. **e**, Stained with anti-LeuM3 for patient 13. Macrophages were scattered within the tumour mass. Bar = 75 μM.

class I antigen expression in the granular cell type, which is reported to have a worse prognosis than the clear cell type (Murphy *et al*., 1965), is intriguing. TIM were significantly fewer in cases showing class I reduction than in those with normal class I expression. Since our studies on TIM subpopulations showed that CD8-positive T cells predominantly infiltrated in most cases, the degree of expression of MHC class I antigen on cancer cells is considered to influence the host immune responsiveness against RCC.

The authors thank Dr T Tanikawa (First Department of Pathology, Niigata University School of Medicine) for his advice on tumour pathology, Drs Y. Matsumoto, Y. Ikarashi (Department of Immunology, Niigata University School of Medicine), Drs Y. Sakata, S. Komatsubara, Y. Kitamura, M. Watanabe (Niigata Cancer Center, Niigata), Dr M. Hiraiwa (Koseiren Sanjo General Hospital, Sanjo), Dr T. Ando (Tsubame Rosai Hospital, Tsubame) and Dr T. Watanabe (Sado General Hospital, Sado) for their assistance and useful advice.

### References

BELLDEGRUM, A., MUUL, L.M. & ROSENBERG, S.A. (1988). Interleukin 2 expanded tumor infiltrating lymphocytes in human renal cell cancer: isolation, characterization and antitumor activity. *Cancer Res*., **48**, 206.

BENACERRAF, B. (1988). Role of MHC gene products in immune regulation. *Science*, **212**, 1229.

BERNARDS, R., SCHRIER, P.I., HOUWELLING, A. & 4 others (1983). Tumorigenecity of cells transformed by adenovirus type 12 by evasion of T-cell immunity. *Nature*, **305**, 776.

BONNARD, C., DEPERMASTER, D.S. & KRAEHENBUHL, J.-P. (1984). The streptavidin-biotin bridge technique: application in light and electron microscope immunocytochemistry. In *Immunolabelling for Electron Microscopy*, Polak, J.M. & Varndell, I.M. (eds) p. 95. Elsevier: Amsterdam.

BROCKER, E.B., STUER, L., BRUGGEN, J., REUTER, D.J., MACHER, E. & SORG, C. (1985). Phenotypic dynamics of tumor progression in human malignant melanoma. *Int. J. Cancer*, **36**, 29.

DAAR, A.S., FUGGLE, S.V., FABRE, J.W., TING, A. & MORRIS, P.J. (1984). The detailed distribution of HLA-A, B, C antigens in normal human organs. *Transplantation*, **38**, 287.

DOYLE, A., MARTIN, W.J., FUNA, K. & 8 others (1985). Markedly decreased expression of class I histocompatibility antigens, protein and mRNA in human small cell lung cancer. *J. Exp. Med*., **161**, 1135.

FOSSATI, G., TARAMELLI, D., BALSARI, A., BOGDANOVICH, G., ANDREOLA, S. & PARMIANI, G. (1984). Primary but not metastatic human melanomas expressing DR antigens stimulate autologous lymphocytes. *Int. J. Cancer*, **33**, 591.

FREED, S.Z., HALPERIN, J.P. & GORDON, M. (1977). Idiopathic regression of Metastasis from renal cell carcinoma. *J. Urol*., **118**, 538.

HEINEMANN, D., SMITH, P.J.B. & SYMES, M.O. (1987). Expression of histocompatibility antigens and characterization of mononuclear cell infiltrates in human renal cell carcinomas. *Br. J. Cancer*, **56**, 433.

JAPANESE UROLOGICAL ASSOCIATION, THE JAPANESE PATHOLOGICAL SOCIETY AND JAPAN RADIOLOGICAL SOCIETY (1983). *General Rules for Clinical and Pathological Studies on Renal Cell Cancer*. Kanehara: Tokyo.

KRADIN, R.L., KURNICK, J.T., LAZARUS, D.S. & 8 others (1989). Tumor-infiltrating lymphocytes and interleukin-2 in treatment of advanced cancer. *Lancet*, **i**, 577.

KROWN, S.E. (1987). Interferon treatment of renal cell carcinoma: current status and future prospects. *Cancer*, **59**, 647.

MÖLLER, P., HERRMANN, B., MOLDENHAUER, G. & MOMBURG, F. (1987). Defective expression of MHC class I antigens is frequent in B-cell lymphomas of high-grade malignancy. *Int. J. Cancer*, **40**, 32.

MOMBURG, F., DEGENER, T., BACCHUS, E., MOLDENHAUER, G., HAMMERLING, G.J. & MÖLLER, P. (1986). Loss of HLA-A,B,C and de novo expression of HLA-D in colorectal cancer. *Int. J. Cancer*, **37**, 179.

MOMBURG, F., HERRMANN, B., MOLDENHAUER, G. & MÖLLER, P. (1987). B-cell lymphomas of high grade malignancy frequently lack HLA-DR, -DP and -DQ antigens and associated invariant chain. *Int. J. Cancer*, **40**, 598.

MOSTOFI, F.K. & DAVIS, C.J. JR (1984). Pathology of tumors of the kidney. In *Cancer of the Kidney*, Javadpour, N. (ed.), p. 15. Thieme-Stratton: New York.

MURPHY, G.P. & MOSTOFI, F.K. (1965). The significance of cytoplasmic granularity in the prognosis of renal cell carcinoma. *J. Urol.*, **94**, 48.

NATALI, P.G., BIOGOTTI, A., NICOTRA, M.R., VIORA, M., MANFREDI, D. & FERRONE, S. (1984). Distribution of human class I (HLA-A,B,C) histocompatibility antigens in normal and malignant tissues of nonlymphoid origin. *Cancer Res.*, **44**, 4679.

PFIZENMAIER, K., BARTSCH, H., SCHEURICH, P. & 4 others (1985). Differential gamma-interferon response of human colon carcinoma cells: inhibition of proliferation and modulation of immunogenicity as independent effects of gamma-interferon on tumour cell growth. *Cancer Res.*, **45**, 3503.

PIONTEK, G.E., TANIGUCHI, K., LIUNGGREN, H. & 4 others (1985). Yak-1 MHC class I variants reveal an association between decreased NK sensitivity and increased H-2 expression after interferon treatment or in vivo passage. *J. Immunol.*, **135**, 4281.

ROSENBERG, S.A., SPIESS, P. & LAFRENIERE, R.A. (1986). A new approach to the adoptive immunotherapy of cancer with tumor-infiltrating lymphocytes. *Science*, **23**, 1318.

ROSENBERG, S.A., LOTZE, M.T., MUUL, L.M. & 10 others (1987). A progress report on the treatment of 157 patients with advanced cancer using lymphokine-activated killer cells and interleukin-2 or high dose interleukin-2 alone. *N. Engl. J. Med.*, **316**, 889.

SAKAI, K., TAKIGUCHI, M., MORI, S. & 5 others (1987). Expression and function of class II antigens on gastric carcinoma cells and gastric epithelia: differential expression of DR, DQ and DP antigens. *J. Natl Cancer Inst.*, **79**, 923.

TANAKA, K., TSSELBACHER, K.J., KHOURY, G. & JAY, G. (1985). Reversal of oncogenesis by the expression of major histocompatibility complex class I gene. *Science*, **228**, 26.

UICC (1987). *TNM Classification of Malignant Tumours*, 4th edn. UICC: Geneva.

VAN DEN INGH, H.F., REUTER, D.J. III, GRIFFIOEN, G., VAN MUIJEN, G.N.P. & FRRONE, S. (1987). HLA antigens in colorectal tumours – low expression of HLA class I antigens in mucinous colorectal carcinomas. *Br. J. Cancer*, **55**, 125.

WANER, J.F. & DENNERT, G. (1982). Effects of a cloned cell line with NK activity on bone marrow transplants, tumour development and metastasis in vivo. *Nature*, **300**, 31.

ZINKERNAGEL, R.M. & DOHERTY, P.C. (1979). MHC-restricted cytotoxic T cells: studies on the biological role of polymorphic major transplantation antigens determining T cell restriction specificity, function and responsiveness. *Adv. Immunol.*, **27**, 51.